# Library of rough hailstone backscattering coefficients at 2.8 GHz

**DOI:** 10.1038/s41597-023-02352-3

**Published:** 2023-07-13

**Authors:** Djordje Mirkovic, Dusan S. Zrnic

**Affiliations:** 1 Cooperative Institute for Severe and High-Impact Weather Research and Operations (CIWRO), Norman, OK, USA; 2 National Severe Storms Laboratory/NOAA (OAR), Norman, OK, USA; 3 The University of Oklahoma, School of Meteorology, School of Electrical Engineering, and Advanced Radar Research Center, Norman, OK, USA

**Keywords:** Natural hazards, Atmospheric science, Electrical and electronic engineering

## Abstract

The rough hailstone scattering library is created to fill the need for results of non-spheroidal hailstone backscattering characteristics. The listed backscattering coefficients at the S-band (2.8 GHZ) are specifically tailored for computing the polarimetric variables. The National Weather Service’s radar operates in this band; hence the library is pertinent to those studying hail in the USA. The library contains several axis ratios and a range of hail sizes from 5 mm to 100 mm. Moreover, hailstone surface roughness is expressed as a percentage of the equatorial diameter. Our computational electromagnetic approach to modeling hailstones is compared with typically employed simpler methods. The polarimetric variables calculated using our models can be compared to observations. Furthermore, we illustrate the potency of the CEM approach to hydrometeors with extreme axis ratios for which simpler methods (i.e., the T-matrix) fail.

## Background & Summary

Calculating the scattering coefficient is an unavoidable process in simulating polarimetric weather radar variables. Scattering coefficients are the basis for the calculation of these variables. Their values are often calculated at a single incident angle and extrapolated to other angles^1^. Most commonly, scattering coefficients are obtained using the Rayleigh approximation for smaller scatterers and the Lorentz-Mie theory for larger scatterers. The T-matrix is among the most often utilized methods for calculating scattering coefficients of sizable spheroidal objects^1^. Some advanced techniques are Discrete Dipole Approximation (DDA)^2^ and Method of Moments (MoM)^3,4^.

The motivation for publishing this library is to bridge the gap between the paucity of naturally occurring hailstone models and spheroidal models. We achieve this by introducing rough hailstone models. Our models are based on the spheroidal shape to which we add surface protuberances.

The idealized spheroidal shape is a common approximation for hail in all the mentioned techniques. Only recently, Jiang *et al*.^2^ modeled naturally occurring hailstones from collected samples. Mirkovic *et al*.^4^ published calculated polarimetric variables from rough spheroidal hail. Herein, we focus on the models used to calculate the polarimetric variables by Mirkovic *et al*.^4^. The library used in^4^ has been published, so it’s readily and widely available to all interested parties. The remainder of the paper is organized to answer questions regarding the hailstone models and their physical and electromagnetic (EM) properties, the coordinate system used, and illustrate the use of the library on examples published in Mirkovic *et al*.^4^. The second section deals with the modeling process and the necessary definition of a coordinate system. The third section explains the library formatting and organization of scattering coefficients. In contrast, the fourth section compares our approach with other accepted models.

## Methods

### Modeling software

The WIPL-D is MoM Surface Integral Equation (MoM-SIE) based commercially available software. The software represents the object as a sum of interconnected infinitesimally thin plates enclosing the boundary. Either side of the plate has electrical properties (permittivity and permeability of material), while the plate’s conductivity may be specified. These “plates” represent the area for which the boundary condition is imposed. The boundary condition determines the induced electric and magnetic currents used to calculate backscattered radiation off the hailstone. Equivalent induced currents are determined for each surface element and approximated by a product of an unknown coefficient and higher-order polynomial basis function. The matrix system is solved to determine the “unknown” coefficients. These coefficients with the basis function are then used to determine the hailstone’s scattered EM field.

MoM and WIPL-D have been validated on many man-made objects^5^, insects, and biota^6,7^. The MoM has been previously used for computational EM (CEM) scattering calculations by hydrometeors. Some early works include modeling hail^8,9^, while some of the latest meteorological applications include predicting scattering by 3D snowflakes^10^. Finally, results published using this library are presented by Mirkovic *et al*.^4^. They show a detailed comparison with results from most previous studies of polarimetric variables in hail.

### The hailstone model

**Fig. 1:**
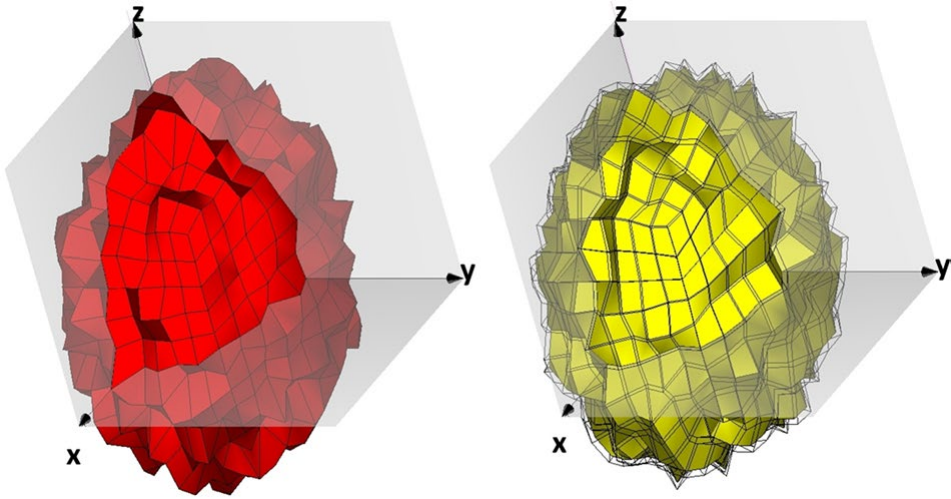
A single and two-layer hail model. Dry hail (single layer) model (left) with electrical permittivity of ice *ɛ*_*r*-*ice*_ = 3.17 − j0.0017. Wet hail (two-layer) model (right) with inner structure (yellow) having ice permittivity and outer layer with permittivity of water ${\varepsilon }_{r-{H}_{2}O}=78.3\,-\,{\rm{j}}12.1$.

All hailstones in the library are simulated at S-band (2.8 GHz) and are differentiated based on the number of layers. Thus “dry” hailstones are represented by a single-layer model, and “wet” hail is represented by a two-layer model (Fig. 1). Dry hailstones are modeled as spheroids with electrical permittivity *ɛ*_*r*-*ice*_ = 3.17 − j0.0017, where their surface represents the aforementioned boundary condition. Wet hailstones are modeled as two concentric spheroids where the inner layer is pure ice (as with dry hailstones), and the outer layer is the water coating. The water coating is based on the Rasmussen formulation of the maximal amount of water prior to shedding^11^. The topology of water coating follows the topology of the ice core.

This shape approximation is introduced to simplify model creation, as each layer must represent a closed surface. Therefore, creating areas or dry spikes that protrude from the water coating would represent complexity in model creation that must be separately treated to ensure models’ regularity in the EM sense. Domain irregularities may result in uncertain scattering outputs.

Each hailstone is defined by its aspect ratio (0.6, 0.7, and 0.8 in the library), and an axis ratio smaller than unity denotes oblate spheroids. The average equatorial diameter of the dry hailstone is given for each hydrometeor in the library (5 to 100 mm). Using these quantities, the dry hailstone’s volume and, therefore, mass can be calculated. In the case of wet hailstones, one must add the mass of the water coating given by the Rasmussen formulation^11^ to obtain the correct mass.

Roughness is added to the hailstone models’ radii as a random number within a predetermined range. In our case, this range is determined in two similar ways, and thus we differentiate two types of roughness in the library. In the first case, “R” type hailstones roughness is a percentage of the equatorial radius. And the second type (“M” type) roughness is a percentage of the radius at each point. This means that “M” type roughness is tapered towards the poles and roughest around the equator. Using these two types of roughness illustrates how different protrusions, easily implemented and controlled, may affect the polarimetric variables. We use roughness of 2, 6, 10, and 14% of the equatorial radius in the library. These values approximate the observed ones published in^4^.

### Coordinate system

Creating the library^12^ in the WIPL-D software requires positioning a hailstone within the WIPL-D software coordinate system. The WIPL-D coordinate system is a right-hand *xyz* system with azimuth angle *φ* defined in the *xOy* plane in the counterclockwise (CCW) direction from *x* to *y*. The elevation angle *θ* is defined as the angle between the *xOy* plane and the *z*-axis. Therefore, the angles of our interest in the WIPL-D coordinate system are 0 ≤ *φ* ≤ 360° and −90 ≤ *θ* ≤ 90° hence we use these the result outputs. The particle rotation for a fixed incident field direction is achieved analogously by keeping the particle fixed and moving the incident field direction over the *φ* and *θ* angles. This is governed by the CEM software, which requires a new simulation for each model change (i.e., rotation). In contrast, the rotation of the incident field can be achieved within a single simulation.

To ensure the highest number of canting angles in the plane of polarization, we position all the hydrometeors in the library with their equatorial plane in the *xOz* plane and the symmetry axis of the original spheroid aligned with the *y*-axis of the WIPL-D coordinate system. Let us consider the two coordinate systems: the fixed hydrometeor coordinate system in which the equatorial plane of the hydrometeor is *x*_*h*_*Oy*_*h*_ and the rotating coordinate system defined by the rotation using the original WIPL-D *φ* and *θ* angles. The rotating coordinate system is defined by the *E*_*φ*_ and *E*_*θ*_ axis (vertical and horizontal polarization vectors) and the *P* axis denoting the direction of the Poynting vector. The *P* axis is always pointed towards the origin of the hydrometeor.

For these two coordinate systems, rotation using *φ* yields the rotation around the y_h_ axis (hydrometeor roll) of the fixed hydrometeor coordinate system as well as canting in the plane of polarization in terms of the Poynting vector. The rotation using *θ* corresponds to rotation around z_h_ axis (pure hydrometeor yaw) for *φ* = 0°, and yaw in terms of the Poynting vector. Whereas the change of *θ* for *φ* ≠ 0°, introduces a combination of roll, yaw, and pitch of hydrometeor in terms of Poynting vector direction.

Rotation of the hydrometeor in this manner introduces nonuniform distribution of orientation points. This is beneficial as the orientation of the hydrometeor dictates that most points are around the poles of the original WIPL-D coordinate system $\theta =\pm \frac{\pi }{2}$ which corresponds to hydrometeor’s canting in the plane of polarization (equatorial plane of hydrometeor perpendicular to the plane of polarization). This nonuniform distribution of orientations is evident if we consider the following. Let us assume that 1° is a sufficient solid angle increment to describe a scatterer. This means that over the full solid angle, we have 41252 increments. Previously, we mentioned that in WIPL-D (as well as other CEM solvers), our angle of interest is $\left(\varphi ,\theta \right)\left(\left(0,2\pi \right),\left(-\frac{\pi }{2},\frac{\pi }{2}\right)\right)$. If we independently apply the same 1° increment over these two angles, we end up with 64800 increments. This is why we chose to orient the hydrometeor with its equatorial plane in the xOz plane of the original WIPL coordinate system, as the nonuniform distribution of orientations allows for the highest number of canting angles within one WIPL-D simulation. In the WIPL-D software, the number of incident angles with 1° increment is 65341 as both 0° and 360° azimuths are considered, and −90° and 90° elevations are considered. This results from the particular CEM software algorithm, which is unimportant for our presentation.

**Fig. 2:**
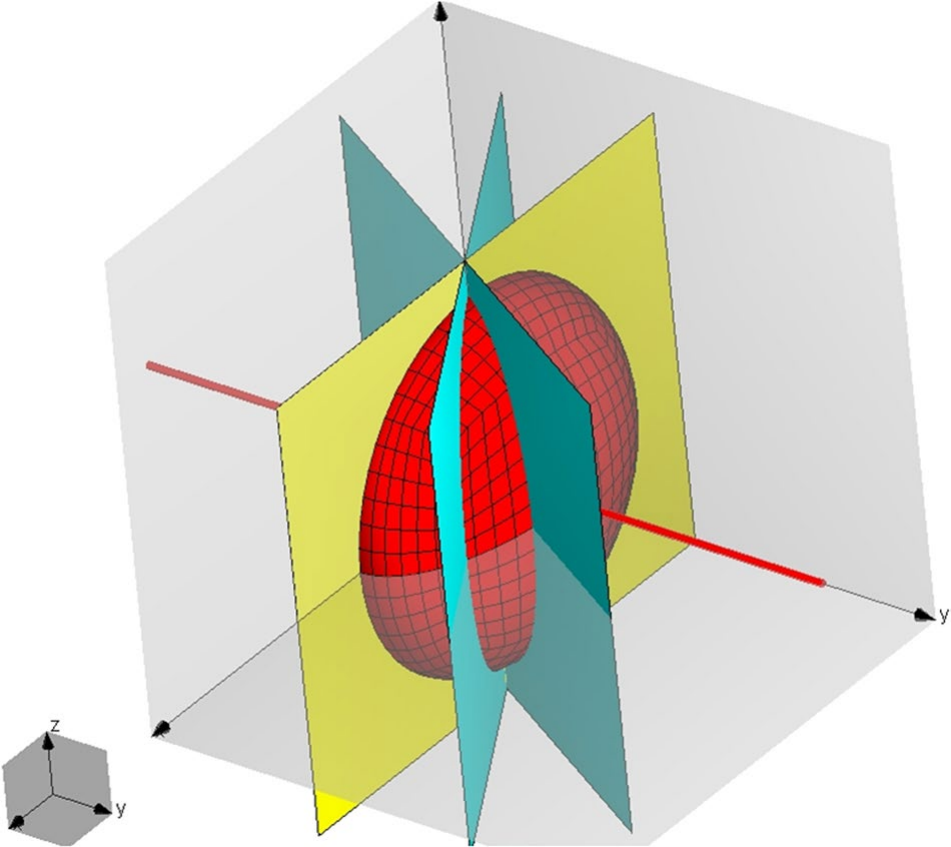
Model in the WIPL-D coordinate system showing the hydrometeor’s symmetry axis (along *y*) and “slicing” planes (blue) with hydrometeor’s horizontal (cardinal) plane (yellow).

Because of rotating the hydrometeor, our backscattering results per simulation are in “slices like a laid down watermelon” (symmetry axis in the *y-*direction) (Fig. 2). This differs from a case whereby the results would be arranged in parallel planes.

**Fig. 3:**
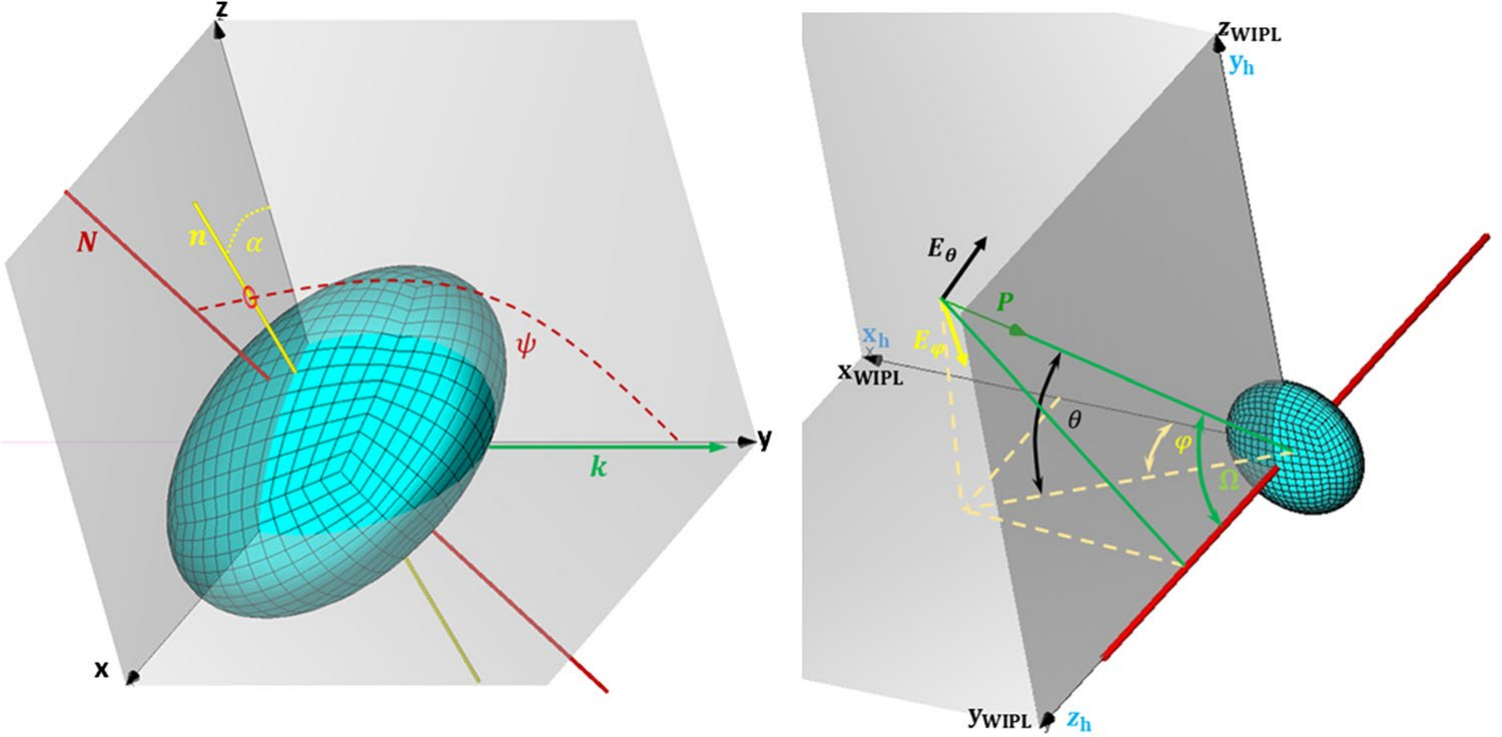
Coordinate systems used. The scatterer-centered coordinate (left) system as defined by Ryzhkov^1^. The Hailstone axis of symmetry is denoted by the red line N; the projection of the symmetry axis to the plane of polarization is the yellow line n, whereas k is the direction of wave propagation. The polarization plane is the xOz plane. Hailstone in the WIPL-D coordinate system (right) subfigure shows three coordinate systems, WIPL-D (x,y,z), hydrometeor (x_h_,y_h_,z_h_), and the coordinate system being rotated (electric field coordinate system *E*_*φ*_, *E*_*θ*_, *P*). Hydrometeor’s equatorial plane is in the xOz plane of the WIPL-D coordinate system. The Poynting vector *P* denotes the direction of propagation, *E*_*φ*_ and *E*_*θ*_ are the polarization vectors (vertical and horizontal) perpendicular to *P*. The azimuth angle *φ* in the WIPL-D coordinate system is in the CCW direction from the x to the y-axis. Elevation *θ* is measured from xOy plane $\theta \left(-\frac{\pi }{2},\frac{\pi }{2}\right)$. Angle Ω is supplementary to the hydrometeor orientation angle *ψ* (i.e., *ψ* = *π*−Ω).

The model orientation (Fig. 3) in terms of the geometry of scattering angles is defined using canting and orientation angle of the scatterer as in^13^. We can calculate canting in the plane of polarization and orientation. Canting in the plane of polarization will be the same as the angle *φ* while determining the orientation *ψ* requires solving the right triangle between the direction of propagation and the axis of symmetry (Fig. 3 right). The orientation angle in terms of WIPL-D angles *ψ* = *π*-arccos[*sin*(*φ*)*cos*(*θ*)]. For special cases $\varphi =\frac{\pi }{2};\frac{3\pi }{2}| \psi =\frac{\pi }{2}$ and $\theta =\frac{\pi }{2};\frac{3\pi }{2}| \psi =\frac{\pi }{2}$.

Information on the particle’s non-spherical shape, embedded in the depolarization scattering elements *s*_*hv*_ and *s*_*vh*_, is coupled with the particle orientation information, which is also related to the difference between the copolar scattering elements *s*_*hh*_ and *s*_*vv*_. If the scatterer’s orientation is collinear with the incident electric fields, its axis ratio can be determined from the intensities of the copolar scattering elements. However, it should be noted that as the actual hydrometeor may be canted (tilted), the inferred axis ratio would be smaller and could be called the apparent axis ratio.

### Direct calculation of polarimetric variables

A large number of hailstone orientations given in the library for each hydrometeor are aimed to alleviate the need for angular moments and the backscattering rule. Angular moments are typically used with the forward operator^14^ to introduce hydrometeor canting. Because the scattering coefficients are available for most orientations, the remaining ones can be obtained by extrapolating the values from the available orientations. Calculating the polarimetric variables using this library is presented in^4^ with formulation for polarimetric variables given by eqs. (1) to (8)^4^.

## Data Records

The library is available for download at NOAA NCEI^12^. The hailstone library consists of almost 1000 data files that store the scattering coefficients. The library covers “dry” (D) and “wet” (W) hailstones with an equatorial diameter ranging from 5 to 100 mm. The equatorial diameter for rough hailstones is the average of the angular diameters in the equatorial plane. In the case of wet hailstone models, the water coating is added to the ice core for which the equatorial diameter is defined. Therefore, the equatorial diameter of the hailstone with water coating will be slightly larger than the value recorded.

The filename is generated in the A##_d###_##.cft format, where the first  defines the type of the hailstone (D or W). In contrast, number signs (#) are used to denote the axis ratio (range 0.1 to 9.9), diameter in mm (5 to 100), and second  denotes the aforementioned type roughness (“R” or “M”) as a percentage of equatorial radius (2 to 14%).

Each scattering coefficients (.cft) file is organized as a tab-separated file with a header. The header contains the frequency for which scattering coefficients are calculated, followed by the scattering coefficients. Table 1 represents how and what values for each scatterer are stored.

**Table 1:** Format of the scattering coefficient library file (.cft).

Frequency ### GHz Header									
φ	θ	Re(s_vv_)	Im(s_vv_)	Re(s_hv_)	Im(s_hv_)	Re(s_vh_)	Im(s_vh_)	Re(s_hh_)	Im(s_hh_)

## Technical Validation

Using MoM software to model man-made objects and hydrometeors, biota, and pyrogenetic particles have been done previously^3,4,6,15^. Furthermore, rigorous testing of WIPL-D and comparisons with other accepted methods for spheroids with various sizes and axis ratios were made. The WIPL-D can be used to determine the T-matrix’s numerical stability, which is known to have convergence issues^16^ in cases of high axis ratios.

To illustrate the application of the WIPL-D in modeling various axis ratios, we made three comparisons. The numerical stability of the model is examined using the scattering coefficients instead of the commonly used radar cross-section (RCS). We accept this unorthodox approach as the convergence issues of the T-matrix for both polarizations appear at an axis ratio of about 3. If we considered the RCS in this comparison, the values would be significantly harder to detect as the *RCS* = 4*π*|*s*_*xx*_|^2^, and the magnitude of the scattering coefficient would mask the convergence issues in V polarization for axis ratios of about 5.

In the first scenario, we model an unrealistically large raindrop (equivolume diameter 10 mm). We consider horizontal (H) and vertical (V) polarization for normal incidence, in which V polarization is parallel to the raindrop’s symmetry axis.

**Fig. 4:**
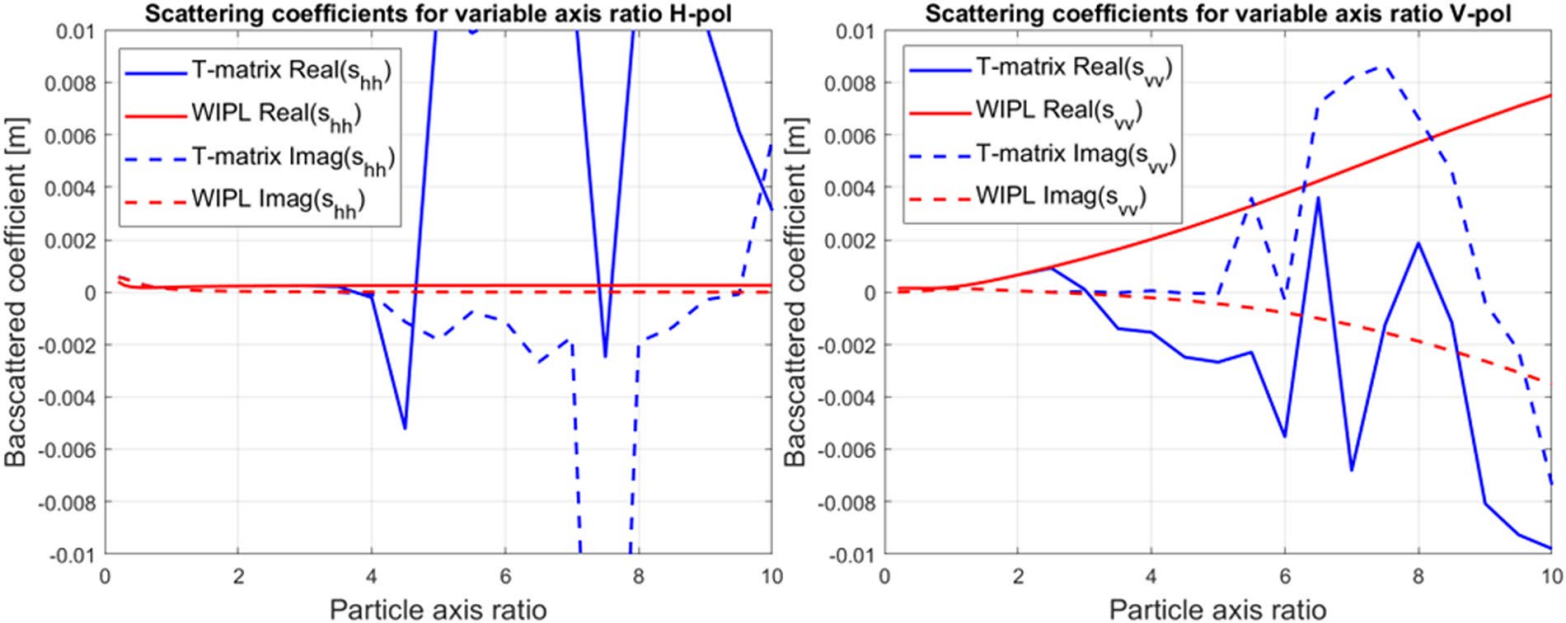
Scattering coefficients for H (left) and V (right) polarization of 10 mm equivolume diameter water (ε_r_ = 78.2 − j12.1) spheroid with axis ratio ranging from 0.2 to 10, at S-band (2.8 GHz). The figure shows the axis ratio at which scattering coefficients calculated by T-matrix become unstable.

Examining the scattering coefficients in Fig. 4, we notice how the real component of the s_hh_ for horizontal polarization becomes unstable for an axis ratio of about 3.5. For vertical polarization, the convergence of T-matrix calculation is compromised at a lower axis ratio of 2.5.

**Fig. 5:**
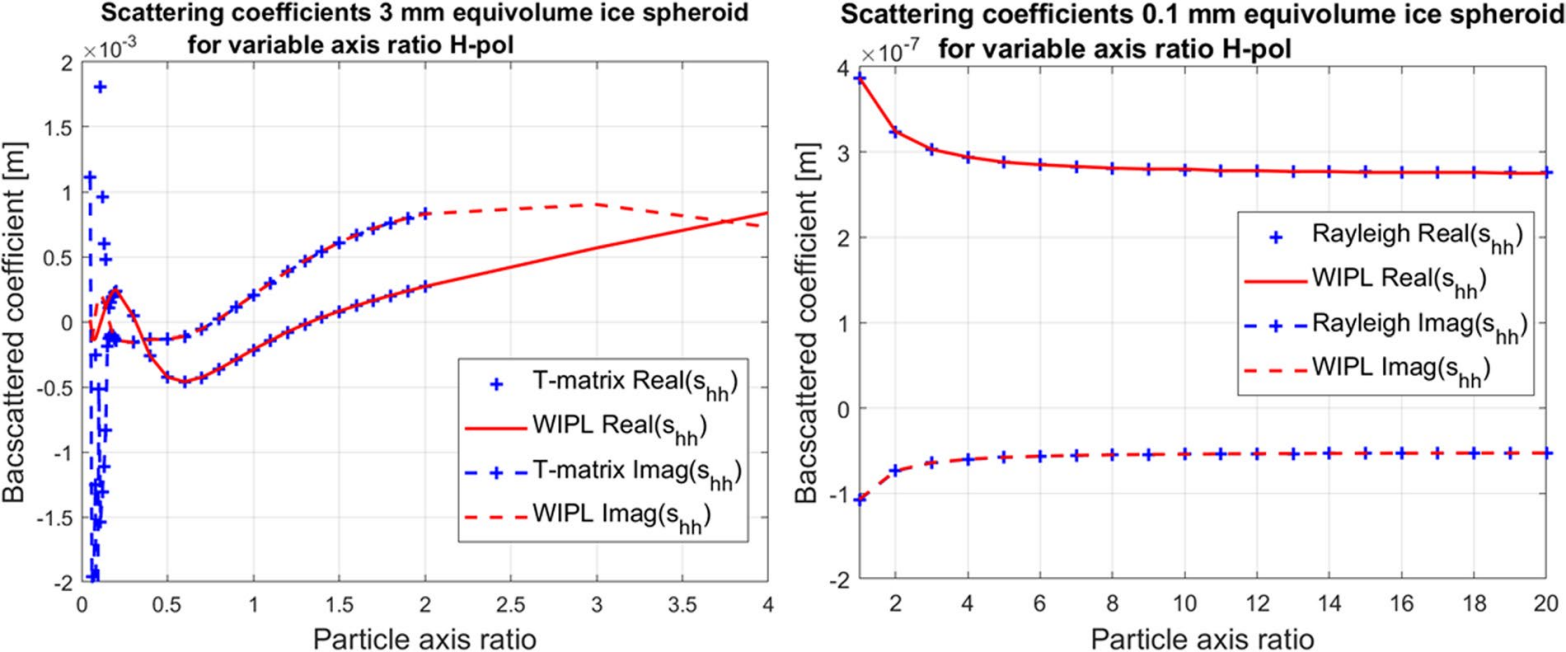
Scattering coefficients for spheroids with varying axis ratio at 90 GHz. A 3 mm equivolume diameter ice (ε_r_ = 3.17 − j9.6) spheroid (left) with an axis ratio ranging from 0.2 to 2. A 0.1 mm equivolume diameter ice spheroid (right) was calculated using Rayleigh approximation due to failure of the T-matrix method.

We use an oblate ice spheroid model with an equivolume diameter of 3 mm to determine if the T-matrix computational method has the lower stability limit dictated by the axis ratio. The axis ratio varies between 0.05 (oblate) and 20 (prolate) to cover lower and upper limits. As the T-matrix we use failed to produce (code failed its internal convergence criteria) any results for prolate spheroids, we chose to use the Rayleigh approximation and thus had to switch to a higher frequency. The frequency for this comparison is changed to W-band (90 GHz). The results are in Fig. 5 (left). For the 3 mm equivolume model and axis ratios higher than two, the T-matrix failed to converge (internal T-matrix convergence). Thus, the blue signs in Fig. 5 (left) end at 2. T-matrix had erratic behavior and did not produce stable results for extremely oblate particles. The axis ratios of 0.2 to 2 perfectly align with the WIPL-D results. To examine behavior for very prolate particles, we had to resort to the Rayleigh approximation and thus decreased particle size to the equivolume diameter of 0.1 mm. These results, shown in Fig. 5. (right), are almost identical.

To further illustrate the effect of roughness on the polarimetric variables, we refer readers to^4^. Examining Fig. 3b,c^4^, we can see how different roughness percentage changes the magnitude squared of s_hh_ to s_vv_ ratio (Z_DR_) of R-type hailstones. Interestingly, changes are most prominent for wet hailstones at resonant sizes. Resonances are easiest to detect as drops of *ρ*_*HV*_ in Fig. 3e,f^4^_._ The behavior of cross-polar scattering can be illustrated from the perspective of |s_vh_|^2^ normalized to |s_hh_|^2^ results, which is L_DR_. The L_DR_ is given in Fig. 3i,j^4^, where roughness introduces deviation of L_DR_’s monotonic increase with size (Fig. 3i^4^). For wet hailstones, the roughness effect is very prominent and seen as very large oscillations of about 15 dB for resonant sizes.

And finally, the database was used to understand better negative differential reflectivity signature (Z_DR_) in hailstorms^4^. These authors used Z_DR_s corresponding to a specific range (50 mm to 65 mm) of water-coated hailstones to speculate about size sorting within the hailstorm. These applications are just a small sample of the many possibilities the database offers.

The database is suitable for radar meteorology applications where a scattering of more realistically modeled hailstones is necessary. Significantly, it can accurately represent the polarimetric variables from hail in supercells, where large and giant hail reside. Its capability to capture especially low differential reflectivity and copolar correlation coefficient (ρ_HV_) may be crucial to explain microphysical processes that simpler models would miss.

The data is available for download at NOAA NCEI^12^ and NSSL’s webpage^17^.

## Usage Notes

Scattering coefficients in the database are stored in the .cft files. Its tabulated format with header makes it easy to access using any programming language or software tool (i.e., Matlab). Results are organized as in Table 1. Coefficients are calculated at 2.8 GHz for dielectric permittivities of ice and water. ε_*r*-*ice*_ = 3.17 − j0.0017 and ${\varepsilon }_{r-{H}_{2}O}=78.3\,-\,{\rm{j}}12.1$. Scattering coefficients are in units of a meter. Additionally, the binary format of the database allows for downloading only the actual scattering matrices in a more compressed format than the .cft text format. We kindly ask the reader to use the data set under the CC-BY license.

## Data Availability

The library data can be accessed in text or binary format. Whereas the library format is described and straightforward for the text files, loading the binary files is best achieved using the MATLAB command “dataBIN = fread(read,[65341,10],‘float’);” where the [65341,10] sets the proper data format (table format). For the ease of access and download in various format database is available in the compressed format at NSSL webpage^17^.
